# The Attenuated Live Yellow Fever Virus 17D Infects the Thymus and Induces Thymic Transcriptional Modifications of Immunomodulatory Genes in C57BL/6 and BALB/C Mice

**DOI:** 10.1155/2015/503087

**Published:** 2015-09-17

**Authors:** Breno Luiz Melo-Lima, Danillo Lucas Alves Espósito, Benedito Antônio Lopes da Fonseca, Luiz Tadeu Moraes Figueiredo, Philippe Moreau, Eduardo Antonio Donadi

**Affiliations:** ^1^Division of Clinical Immunology, Department of Medicine, Ribeirão Preto Medical School, University of São Paulo, Avenida Bandeirantes 3900, 14049-900 Ribeirão Preto, SP, Brazil; ^2^Commissariat à l'Energie Atomique et aux Energies Alternatives, Institut des Maladies Emergentes et des Thérapies Innovantes, Service de Recherches en Hémato-Immunologie, Hôpital Saint-Louis, 1 avenue Claude Vellefaux, Bâtiment Lailler, 75475 Paris Cedex 10, France; ^3^Université Paris-Diderot, Sorbonne Paris-Cité, UMR E5, Institut Universitaire d'Hématologie, Hôpital Saint-Louis, 1 avenue Claude Vellefaux, 75475 Paris Cedex 10, France; ^4^Virology Research Center, Ribeirão Preto Medical School, University of São Paulo, Avenida Bandeirantes 3900, 14049-900 Ribeirão Preto, SP, Brazil

## Abstract

Thymus is involved in induction of self-tolerance in T lymphocytes, particularly due to Aire activity. In peripheral tissues, Treg cells and immunomodulatory molecules, like the major histocompatibility complex (MHC) class Ib molecules, are essential for maintenance of autotolerance during immune responses. Viral infections can trigger autoimmunity and modify thymic function, and YFV17D immunization has been associated with the onset of autoimmunity, being contraindicated in patients with thymic disorders. Aiming to study the influence of YFV17D immunization on the transcriptional profiles of immunomodulatory genes in thymus, we evaluated the gene expression of *AIRE, FOXP3, H2-Q7* (Qa-2/HLA-G), *H2-T23* (Qa-1/HLA-E), *H2-Q10*, and *H2-K1* following immunization with 10,000 LD_50_ of YFV17D in C57BL/6 and BALB/c mice. The YFV17D virus replicated in thymus and induced the expression of *H2-Q7* (Qa-2/HLA-G) and *H2-T23* (Qa-1/HLA-E) transcripts and repressed the expression of *AIRE* and *FOXP3*. Transcriptional expression varied according to tissue and mouse strain analyzed. Expression of *H2-T23* (Qa-1/HLA-E) and *FOXP3* was induced in thymus and liver of C57BL/6 mice, which exhibited defective control of viral load, suggesting a higher susceptibility to YFV17D infection. Since the immunization with YFV17D modulated thymus gene expression in genetically predisposed individuals, the vaccine may be related to the onset of autoimmunity disorders.

## 1. Introduction

Autoimmune disorders have a complex and multifactorial etiopathogenesis, arising from an imbalance between the mechanisms involved in maintenance of immune tolerance and those related to the generation of an effective immune response [1]. The thymus is the primary lymphoid organ related to establishment of central tolerance and related to functional and phenotypical maturation of thymocytes [2]. The main events in central tolerance of thymocytes are the positive and negative selections [2, 3], characterized, respectively, by the maintenance of cortical double-positive thymocytes that recognize with low affinity/avidity self-antigens presented by self-histocompatibility molecules and by the apoptotic clonal deletion of medullary thymocytes that recognize self-antigens with high affinity/avidity [2–5].

The major regulator of negative selection is the* AIRE* (autoimmune regulator) gene [2, 6], which imposes the expression of clusters of hundreds of tissue-related (TRAs) genes from peripheral tissues inside the thymic medulla, by the phenomenon known as Promiscuous Gene Expression (PGE) [6–8]. Therefore, PGE represents a remarkable feature to ensure the exposure of the majority of tissue antigens from the body in thymic medulla to prevent autoimmunity [2, 6]. During negative selection, subpopulations of thymocytes can differentiate in natural regulatory T (nTregs) cells by the expression of FOXP3 during clonal deviation [9, 10]. Both Aire and FOXP3 are transcription factors essential to prevent autoimmunity and to maintain immunological homeostasis [2, 11, 12]. Single nucleotide variations in these genes are related to severe systemic autoimmune syndromes, such as APECED (autoimmune polyendocrinopathy-candidiasis-ectodermal dystrophy) [13] and IPEX (immunodysregulation polyendocrinopathy enteropathy X-linked syndrome) [12, 14].

Variations in expression of* AIRE* and* FOXP3* may produce changes in thymic function and therefore may influence the systemic immune responses [2, 15, 16]. The thymus is a common target organ during cancer [17, 18] and infectious diseases [19–21], undergoing relevant changes in function and morphology. Viral infections can invade and spread into thymus influencing positive and negative selection, facilitating the appearance of potentially autoreactive thymus-derived immature CD4^+^CD8^+^ T cells [21]. In genetically predisposed individuals, viruses can also trigger events that may promote the onset of autoimmune disorders with an overstimulation of the immune system, which may allow molecular mimicry between pathogen associated-antigens and host antigens [22].

The Yellow Fever Virus (YFV) is a member of the Flaviviridae family, exhibiting a positive-sense single-stranded RNA, which infects humans and other vertebrates [23]. This virus is mainly transmitted by bites of female mosquitoes (mostly from genus* Aedes*), occurring in endemic areas of tropical regions [24], causing a form of hemorrhagic fever characterized by headache, high fever, nausea, myalgia, jaundice, cardiovascular complications, and multiple organ failure in severe cases [23, 25, 26]. There are no antiviral therapies against YFV; however, since 1945, a live virus attenuated vaccine (YFV17D) is available in two substrain forms: a 17DD and 17D204 [23, 27].

YFV17D immunization is considered to be a safe and effective method for the protection against Yellow Fever Virus infection [28]. However, severe adverse effects related to YFV17D immunization as the Yellow Fever Virus Associated Viscerotropic Disease (YEL–AVD) and the Yellow Fever Virus Associated Neurotropic Disease (YEL–AND) have been described [23]. Interestingly, YEL-AVD cases have been associated with deaths in patients with thymic dysfunction caused by thymomas, thymectomy, myasthenia gravis, or DiGeorge syndrome [29]. Thymic disorders are one of the most relevant risk factors associated with deaths after YFV17D immunization, highlighting the involvement of thymus-derived cells in controlling the viral immune responses after immunization [29, 30].

The thymus is considered to be a primary site for expression of immunomodulatory molecules such as Aire and FOXP3 [2, 11]. Interestingly, the constitutive expression of nonclassical class I major histocompatibility complex (MHC Ib) molecules has been reported in several subpopulations of thymic cells [31–33]. Human MHC Ib molecules, such as HLA-G and HLA-E, are associated with the regulation of immune responses in the periphery by interacting with inhibitory receptors [34, 35]. These molecules exhibit restricted peripheral tissue distribution; they are not involved in antigen presentation and do not show high rate of polymorphisms [34, 35].

In mice, two molecules have been described to be functional homologues of HLA-G and HLA-E, that is, Qa-2 and Qa-1, respectively [31, 36]. Similarly to humans, Qa-2 and Qa-1 molecules are involved in the regulation of immune responses, by inhibiting maternal NK cell-mediated lysis [35–37], suppression of CD4^+^ T cell and NK cell responses through a preferential interaction of Qa-1 with inhibitory CD94/NKG2A receptors [38, 39], and induction of CD8^+^ regulatory T cells restricted to antigens presented by Qa-1 molecules [38, 39]. Qa-2 and Qa-1 are encoded by* H2*-*Q7/H2-Q9* and* H2-T23* genes, respectively, both located at the histocompatibility complex-2 in chromosome 17 [40, 41].

Due to the immunomodulatory properties of MHC Ib molecules and due to the relevance of Aire and FOXP3 in self-tolerance, it is hypothesized that changes in expression of these molecules in thymus during antiviral responses could be related to (i) alterations in thymic function, resulting in a defective PGE and negative selection, (ii) modifications in the regulatory T cell repertoire, and (iii) appearance of autoimmune disorders [7, 16, 19]. In addition, it has been reported that live virus attenuated vaccines are associated with autoimmune diseases [42–44]. Particularly, immunization with YFV17D has been related to several cases of longitudinal myelitis and Kawasaki disease [45, 46].

Based on these evidences, we hypothesized that the YFV17D immunization could promote alterations in thymic function, influencing the transcriptional profile status of immunomodulatory genes such as* AIRE*,* FOXP3*,* H2-Q7* (Qa-2/HLA-G), and* H2-T23* (Qa-1/HLA-E) in thymus and in relevant peripheral organs such as the spleen and liver. Also, we hypothesized that the genetic background of different murine strains (C57BL/6 and BALB/c) could contribute to differential modulation in gene expression, therefore influencing the patterns of susceptibility/resistance during immune response to YFV17D infection.

## 2. Material and Methods

### 2.1. Animals

Young adult C57BL/6 and BALB/c mice were obtained from the local animal facility and maintained in isolated cages, provided with 0.45 *μ*m pore size air filter. Experimental procedures followed ethical guidelines under strict guidance and approval by the University of São Paulo Ethics Committee for Animal Experimental Research (Protocol number 180/2009).

### 2.2. Virus

The Yellow Fever Virus vaccine substrain 17DD (YFV17D) used for mouse infection was produced in the Virology Research Center, University of São Paulo, Ribeirão Preto, Brazil. Briefly, virus stocks were obtained from brains of intracerebrally infected newborn mice. Mouse brains were macerated in phosphate buffered saline (PBS) (dilution 1 : 20 wt/vol) and centrifuged at 10000 RPM for 10 min at 4°C, and supernatants were stored at −80°C.

### 2.3. Infection of Mice by YFV17D and Negative Controls

Fifty 8-week-old females of wild-type young adult C57BL/6 and BALB/c mice were infected intraperitoneally (IP) with 10,000 LD_50_ of YFV17D in a volume of 200 *μ*L/PBS 1x pH 7.4 solution at Day 0. The animals were monitored daily and most of them developed encephalitis signs such as hind limb paralysis, tremors, muscle weakness, ruffled pile, and difficulty to feed. The animals were killed at Days 1, 5, 7, 15, and 30 after inoculation (a.i.). Negative control mouse groups (*N* = 50) were inoculated IP with PBS 1x pH 7.4 and killed at Days 1, 5, 7, 15, and 30 a.i. and their tissues were used for comparisons with those of YFV17D-infected animals. Tissue samples from thymus (both lobes), spleen, and liver (posterior extremity and right lobe, resp.) were surgically collected from at least 5 different animals for each Day a.i., washed in saline solution, and then processed without separation of hematopoietic cells and parenchyma.

### 2.4. RNA Extraction

After tissue isolation, total RNA was obtained by maceration of each tissue in TRizol reagent using a Potter homogenizer, according to the manufacturer's instructions (Invitrogen, Carlsbad, CA), and treated with DNAse (deoxyribonuclease I amplification grade, Invitrogen). RNA integrity was checked by the presence of the 28S and 18S bands in 1.5% denaturing agarose gel, and purity of RNA was accessed by UV-Vis spectrophotometer (NanoDrop 2000, Thermo Scientific, MA), regarding amount of protein and organic compounds in the samples. Only RNAs exhibiting 260/230 and 260/280 ratios of ~2.0 ratio were used for quantitative PCR experiments. Total RNA was reverse transcribed to cDNA using the High Capacity cDNA Transcription Kit (Applied Biosystems, Foster City, CA), following the manufacturer's instructions. cDNA amplification was initially carried out in a total volume of 25 *μ*L, corresponding to 500 *η*g of the initial RNA.

### 2.5. Detection of the Viral Load by Quantitative Real-Time PCR

The detection of the viral load and quantification was performed by quantitative real-time PCR (qPCR) method, using specific primers (forward:* 5-GTGACGGCTCTGACCAT-3*; reverse:* 5-ATGCAGTGAGCTGAGTA-3*) for the viral envelope gene (*E*)* protein*. In brief, about 150 *η*m of each primer; 5 *μ*L SYBR Green PCR Kit (Qiagen, Valencia, CA); and 3 *μ*L of cDNA and nuclease-free water (Sigma-Aldrich, St. Louis, MO) in sufficient quantity for a final volume of 10 *μ*L per reaction were used. The number of genome copies was obtained by absolute quantification using a serial dilution of envelope (*E*)* protein* gene, previously cloned in a plasmid (InsTAclone, Fermentas, Burlington, ON), to generate a standard curve to be used as reference values.

### 2.6. Analysis of Expression by Quantitative Real-Time PCR

The expression of* H2-Q7* (Qa-2/HLA-G),* H2-T23* (Qa-1/HLA-E),* H2-Q10* (Qa10),* H2-K1*,* AIRE,* and* FOXP3* genes was assessed by qPCR, using TaqMan Probe-Based Gene Expression Analysis (Applied Biosystems) in a total volume of 10 *μ*L, containing 75 *η*g total RNA, 5 *μ*L TaqMan PCR Universal Master Mix (Applied Biosystems), and 0.5 *μ*L TaqMan Gene Expression Assays. An ABI System Sequence Detector 7500 (Applied Biosystems) was used with the following regimen of thermal cycling: stage 1: one cycle for 2 minutes at 50°C; stage 2: one cycle for 10 minutes at 95°C; stage 3: forty cycles for 15 seconds at 95°C, followed by the last cycle for 1 minute at 60° and 25 seconds at 72°C. Gene expression was normalized relatively to the TaqMan endogenous controls (Applied Biosystems), using geometric mean* glyceraldehyde-3-phosphate dehydrogenase (GAPDH)* and *β-actin* genes. The relative quantification of transcript levels was performed by the comparative 2^−ΔΔCt^ method. Each sample was tested in triplicate. The TaqMan Inventoried Assays and TaqMan endogenous control references are listed as follows:* AIRE*: Mm00477461_m1;* FOXP3*: Mm00475162_m1;* H2-Q7* (Qa-2/HLA-G): Mm00843895_m1;* H2-T23* (Qa-1/HLA-E): Mm00439246_m1;* H2-Q10* (Qa10): Mm01275264_m;* H2-K1*: Mm02342236_gH;* GAPDH*: 4352339E;* ACTB*: 4352341E.

Statistical analysis was performed using nonparametric tests according to the number of studied groups: (i) two groups: Mann-Whitney two-tailed; (ii) three or more groups: Kruskal-Wallis followed by Dunn's multiple comparison. Values of *P* < 0.05 were considered to be statistically significant. All statistical tests were performed with the GraphPad Prism software version 5.0 (GraphPad Software Inc., San Diego, CA).

## 3. Results and Discussion

### 3.1. Detection of YFV17D Viral Load in Harvested Tissues

**Figure 1:**
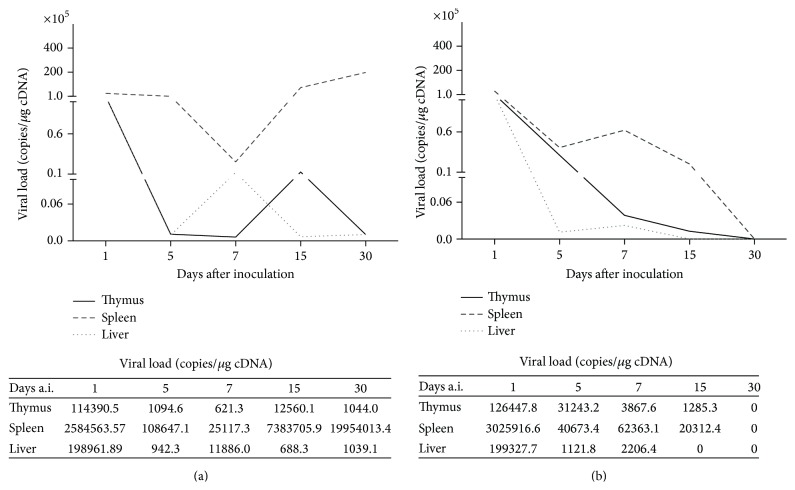
Detection of YFV17D viral load in harvested tissues of (a) C57BL/6 and (b) BALB/c mice. Representative of geometric mean values of viral load (copies/1 *μ*g cDNA) measured by qPCR.

First, we evaluated if the primary and secondary immune system organs could be targeted during YFV17D immunization. Samples of thymus, liver, and spleen harvested at Days 1, 5, 7, 15, and 30 a.i. were tested by qPCR for virus detection and quantification of viral load. In both mice strains, it was observed that the YFV17D could infect the thymus and replicate in this organ until Day 15 a.i. (Figures 1(a) and 1(b)). Increased amount of viral copies in the spleen of C57BL/6 animals was observed throughout infection (Figure 1(a)). Compared with BALB/c mice, the C57BL/6 strain showed increased viral load in all tissues analyzed and was not able to eliminate the infection in spleen at Day 30 a.i. (Figure 1(a)).

It has been reported that YFV17D infects and replicates in immunocompetent cells, such as dendritic cells (DCs), monocytes, and macrophages [23, 47]. In this study, the virus was able to infect the thymus, the liver, and the spleen. It is hypothesized that the interaction of the viral envelope (E) protein with the specific intercellular adhesion molecule-3-grabbing nonintegrin (SIGN molecules) present on the surface of DCs (CD209) and lymphocytes (CD209L) may allow virus internalization and cell infection [47]. Like other infectious agents, including HIV (Human Immunodeficiency Virus), protozoan parasites, and fungi, which have been reported to infect the thymus [19], we also reported that despite the existence of the blood-thymic barrier, the intraperitoneal inoculation of 10,000 LD_50_ of YFV17D in C57BL/6 and BALB/c mice infected the thymus in both strains.

Despite the absence of studies involving the characterization of immune response against YFV17D in C57BL/6 and BALB/c strains, we hypothesized that the differences in the genetic background of these two murine strains could influence the immune response pattern during viral infection. This hypothesis is supported by the fact that the signs of infection, such as hind limb paralysis, tremors, muscle weakness, ruffled pile, and difficulty to feed observed from Days 7 to 30 a.i. in both mice strains, were more pronounced in BALB/c when compared to C57BL/6 mice (data not shown). Additionally, the morphological analysis of internal organs in immunized animals revealed liver and spleen hypertrophy at Day 7 a.i in BALB/c mice compared to C57BL/6 and to control groups (see Supplemental data I in Supplementary Materials available online at http://dx.doi.org/10.1155/2015/503087). These results suggest that BALB/c mice could perform a more robust antiviral response against YFV17D that could reflect in less pronounced morbidity and in a better control of the viral load.

### 3.2. Influence of YFV17D Immunization in the Gene Expression of Immunomodulatory Molecules in Thymus

**Figure 2:**
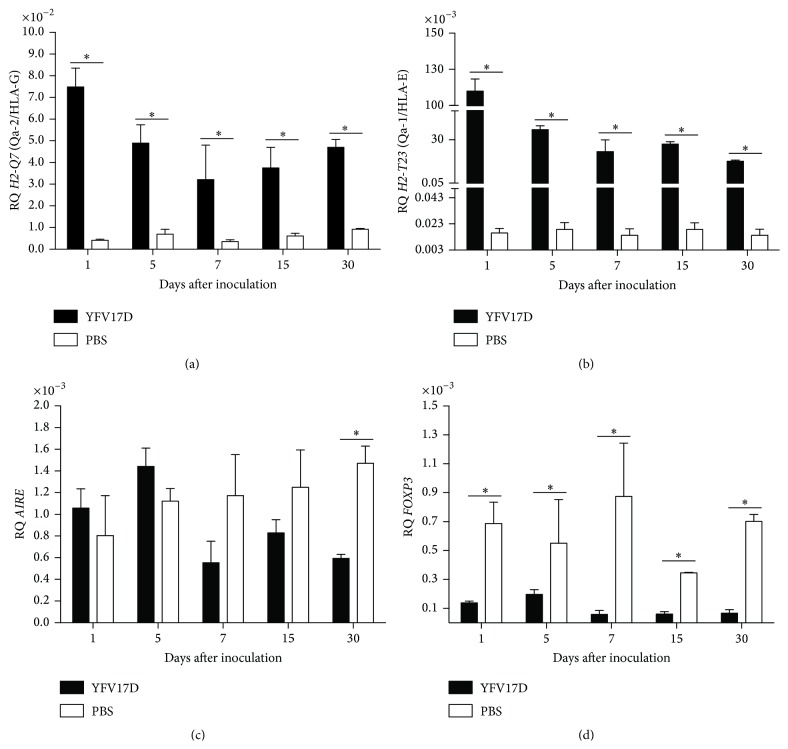
Gene expression of immunomodulatory molecules: (a)* H2-Q7* (Qa-2/HLA-G); (b)* H2-T23* (Qa-1/HLA-E); (c)* AIRE*; and (d)* FOXP3* in thymus of C57BL/6 mice inoculated with 10.000 LD_50_ of YFV17D or PBS. RQ = relative quantification representative of geometric mean values of 2^−ΔΔCt^. Tissue samples were obtained in triplicate from different animals for each treatment. Each experiment was independently performed at least three times. Statistical analysis was performed using the nonparametric test Mann-Whitney two-tailed. Values close to the level of significance (*P* < 0.05) are marked with *∗*.

**Figure 3:**
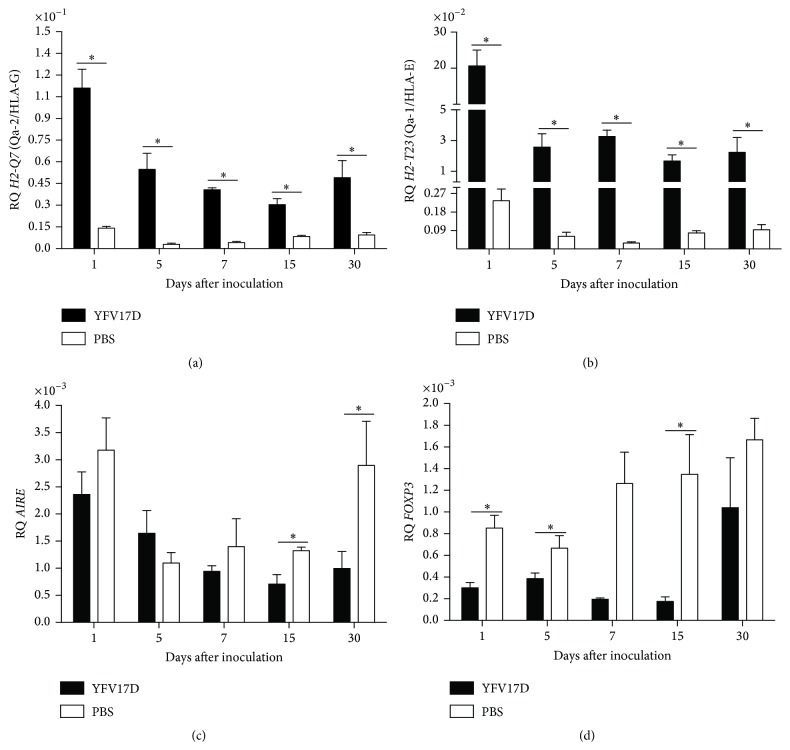
Gene expression of immunomodulatory molecules: (a)* H2-Q7* (Qa-2/HLA-G); (b)* H2-T23* (Qa-1/HLA-E); (c)* AIRE*; and (d)* FOXP3* in thymus of BALB/c mice inoculated with 10.000 LD_50_ of YFV17D or PBS. RQ = relative quantification representative of geometric mean values of 2^−ΔΔCt^. Tissue samples were obtained in triplicate from different animals for each treatment. Each experiment was independently performed at least three times. Statistical analysis was performed using the nonparametric test Mann-Whitney two-tailed. Values close to the level of significance (*P* < 0.05) are marked with *∗*.

Since YFV17D could be detected in thymus, we evaluated the influence of YFV17D immunization on the transcription of immunomodulatory genes in thymi of C57BL/6 and BALB/c mice. The expression of immunomodulatory genes in samples of thymus of YFV17D-infected mice was significantly modified in both strains compared to mice control group. In thymi of YFV17D-treated C57BL/6 mice a transcriptional activation of MHC class Ib genes was observed. Transcripts for* H2-Q7* (Qa-2/HLA-G),* H2-T23* (Qa-1/HLA-E) (Figures 2(a) and 2(b), resp.), and* H2-Q10* (Qa10) (Supplemental data II) were significantly increased from Day 1 a.i. and at all analyzed days compared to PBS-treated mice control group (*P* < 0.05). Differently, a significant transcriptional repression was observed for* AIRE* and* FOXP3* genes in thymi of YFV17D-treated mice compared to control group (*P* < 0.05) (Figures 2(c) and 2(d), resp.). YFV17D-treated BALB/C mice showed similar transcriptional profiles observed for C57BL/6, with significant increased transcripts levels for* H2-Q7* (Qa-2/HLA-G) and* H2-T23* (Qa-1/HLA-E) (Figures 3(a) and 3(b), resp.) and downregulation in* AIRE* and* FOXP3* transcription compared to PBS-treated mice control group (*P* < 0.05) (Figures 3(c) and 3(d), resp.). No differences in* H2-Q10* (Qa10) transcription were observed (Supplemental data II).

These results agree with evidences showing that several pathogens can infect and replicate in thymus influencing its function [19]. It is known that thymus could act as a site of infection during immune responses, where mature T cells are recruited back to thymus in response to intrathymic cytokine (IFN-*γ*) and chemokine (CXCL9, CXCL10, and CXCR3) production during infection [48]. Additionally, several infectious agents are able to modulate the expression of nonclassical MHC class I molecules, promoting the escape of immunosurveillance in peripheral tissues [35, 49–51].

In humans, YFV17D induces the transcription of sets of genes related to innate immunity, including several Toll-like receptors (TLRs), type I IFNs, KIR2DL3, and KIR2DL4 [52]. Additionally, YFV17D promotes the maturation and activation of DCs by the recognition of TLR2, TLR7, TLR8, and TLR9, resulting in a dose-dependent response of proinflammatory cytokines, such as IL-6, IL-1*β*, TNF-*α*, MCP-1, IP-10, and INF-*α* and INF-*β* [53, 54]. YFV17D also stimulates the differentiation of YFV17D-specific CD8^+^ T and CD4^+^ T cells [23] that can be found from Day 7 a.i.; from Day 15 these cells begin to express the receptor for IL-10 and to secrete IFN-*γ* [55].

The transcriptional activation of nonclassical MHC class I* H2-Q7* (Qa-2/HLA-G) and* H2-T23* (Qa-1/HLA-E) in thymus during YFV17D immunization may be attributed to the increased production of proinflammatory cytokines IL-6, IL-1*β*, and TNF-*α* and also INF-*α* and INF-*β* released in thymus or present in bloodstream, during the early events of innate immune response, and persisting at high levels during the adaptive immune response. Additionally, production of IL-10 and TGF-*β* is increased after Day 15 a.i. in YFV17D-immunized patients [55]. Reportedly, these cytokines are related to the induction of nonclassical MHC molecules, such as HLA-G [34, 35].

Considering that the thymus (i) is the primary site of expression of MHC Ib molecules, related to functional maturation of medullary thymocytes, as described for Qa-2 [33, 56], and (ii) plays a relevant role on positive selection of subpopulations of CD8^+^ T cells and regulatory CD8^+^ T cells restricted to recognition of antigens presented by Qa-1 [57, 58] and HLA-E [59], we believe that the transcriptional activation of MHC Ib genes after YFV17D infection could influence the thymic function by altering thymocyte differentiation, possibly by increasing the rate of medullary Qa-2^+^ thymocyte emigration and Qa-1-restricted T cells selection, thus influencing the antiviral response in peripheral tissues.

Additionally, since MHC Ib are related to the modulation of immune system by interaction with inhibitory receptors, such as CD94/NKG2A for HLA-E/Qa-1 [58, 60] and ILT-2/ILT-4 for HLA-G [34, 35], the upregulation of MHC Ib expression in thymus by YFV17D could be related to an increased thymic immunoregulatory network to maintain thymic microenvironment and functionality during antiviral response. Particularly, the upregulation of Qa-1 expression could enhance regulatory mechanisms by the interaction with CD94/NKG2A receptor, promoting inhibition of CD8^+^ T and NK cells cytotoxicity and increasing the regulatory activity and anti-inflammatory cytokine production by Qa-1-restricted suppressor CD8^+^ T cells [61, 62].

The transcriptional repression observed for* AIRE* and* FOXP3* genes in thymus after YFV17D immunization was an interesting finding. Although there are no evidences relating that viral infections can modulate the expression of* AIRE* in human or murine thymus, we hypothesize that the inhibition of this gene during the YFV17D immunization could contribute to the onset of autoimmune manifestations associated with vaccination. We raised this hypothesis due to several evidences suggesting the onset of autoimmune diseases as a result of viral infections and immunizations [44, 46] and, more particularly, due to the important role of* AIRE* in the TRA expression regulation during thymic selection [2, 7]. Decreased levels of* AIRE* may imply a defective negative selection, therefore releasing autoreactive clones of T cells to peripheral tissues [2, 7].

FOXP3 expression decreases during acute infection caused by herpes simplex 1/2 [63] and by West Nile viruses [64], being associated with the aggravation of infection, increasing the mortality rate and increasing viral load in infectious site. Similarly, the ratio between the frequency of effector T cells and regulatory T cells plays an important role on the immunopathogenesis of infections caused by flaviviruses, as reported for dengue [65]. We speculate that the transcriptional repression of* FOXP3* in thymus after administration of YFV17D could contribute to higher severity pathogenic effects associated with the vaccine, as observed for BALB/c mice in the present study.

Aire regulates the induction and frequency of the repertoire of subpopulations of natural regulatory T cells in thymus by unknown mechanisms [66, 67]. We believe that the decreased* AIRE* expression after YFV17D immunization could influence the expression of* FOXP3* and consequently the frequency of nTregs. The diminished frequency of nTreg cells associated with a defective negative selection, due to low levels of* AIRE*, could be an additional evidence for the onset of autoimmunity after immunization with YFV17D.

### 3.3. Transcriptional Profiles of Immunomodulatory Genes during YFV17D Immunization

**Figure 4:**
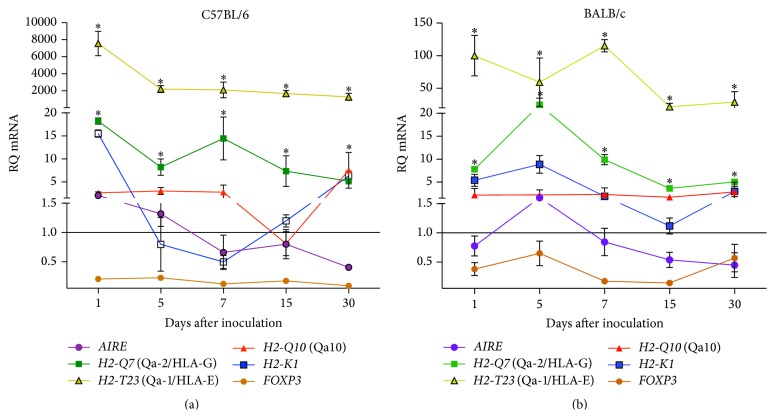
Transcriptional profiles of immunomodulatory genes in thymus of (a) C57BL/6 and (b) BALB/c mice inoculated with 10.000 LD_50_ of YFV17D. RQ = relative quantification representative of geometric mean values of 2^−ΔΔCt^. RQ values expressed as fold change relative to control group values (baseline = 1). Statistical analysis was performed using the nonparametric test Kruskal-Wallis Dunn's multiple comparison. Values close to the level of significance (*P* < 0.05) are marked with *∗*.

**Figure 5:**
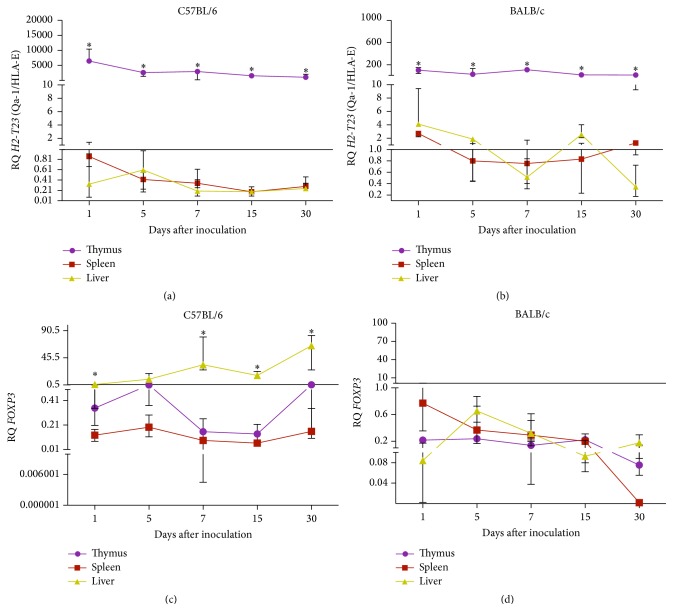
Transcriptional profiles of* H2-T23* (Qa-1/HLA-E) ((a) and (b)) and* FOXP3* ((c) and (d)) in harvested tissues of C56BL/6 and BALB/c mice inoculated with 10.000 LD_50_ of YFV17D. RQ = relative quantification representative of geometric mean values of 2^−ΔΔCt^. RQ values expressed as fold change relative to control group values (baseline = 1). Statistical analysis was performed using the nonparametric test Kruskal-Wallis Dunn's multiple comparison. Values close to the level of significance (*P* < 0.05) are marked with *∗*.

To understand the effect of YFV17D immunization in the transcription of immunomodulatory genes, we characterized the transcriptional profiles of these genes in thymus and peripheral tissues (liver and spleen) of YFV17D-treated mice, comparing the profiles between C57BL/6 and BALB/c strains. After YFV17D immunization, the thymic expression of MHC class Ib transcripts, particularly* H2-T23* (Qa-1/HLA-E) and* H2-Q7* (Qa-2/HLA-G), was increased when compared to MHC class Ia,* H2-K1* transcripts (*P* < 0.05, for each comparison) for both mouse strains. Decreased expression of* AIRE* and* FOXP3* was also observed compared to all genes analyzed (Figures 4(a) and 4(b)).* H2-T23* (Qa-1/HLA-E) was the most widely expressed gene in thymus throughout the immunization, and the thymus was the tissue which most expressed this gene, compared to liver and spleen, in both strains analyzed (*P* < 0.05) (Figures 5(a) and 5(b)). No significant differences were observed for other genes in all studied tissues.

The role of Qa-1 in antiviral immunity has been demonstrated in several viral infections. Increased levels of Qa-1 have been reported in mice infected with Herpes Simplex Virus [62] and cytomegalovirus, when compared to classical MHC class I (H2-D) molecules in peripheral tissues [68]. Particularly, viral infections caused by other flaviviruses, as observed for Japanese Encephalitis Virus, promoted the upregulation of Qa-1 expression in a type 1 IFN-dependent manner, independently of NF-kB pathway [69].

We demonstrated that YFV17D immunization promotes a significant upregulation of Qa-1 in thymus in higher levels than in other peripheral tissues, as liver and spleen, evidencing this central lymphoid organ as a primary expression site of MHC Ib during YFV17D infection. Interestingly, during YFV17D immunization in both strains we observed decreased levels of classical MHC class I gene (*H2-K1*), typically involved in antiviral responses [70, 71], compared to MHC Ib genes (Figures 4(a) and 4(b)). We believe that low levels of* H2-K1* transcripts, as observed in this study, could be related to a viral escape mechanism of immunosurveillance acting in a transcriptional regulation level, contributing to a reduced antigen processing and presentation, as described at molecular level [72].

It is known that the blockade of Qa-1 during acute and chronic antiviral responses enhances the cytolytic activity of effector CD8^+^ T cells, the repression of PD-1 (programmed cell death-1), and the upregulation of activating receptor NKG2D [61]. We speculate that this molecule and its human counterpart HLA-E could play a relevant role during antiviral immunity against YFV and other flaviviruses.

**Figure 6:**
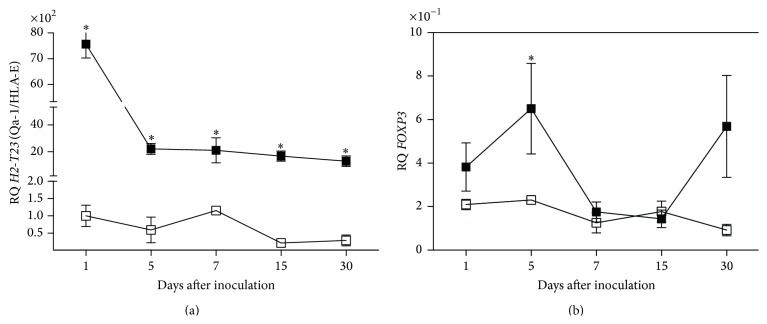
Comparison of transcriptional profiles of (a)* H2-T23* (Qa-1/HLA-E) and (b)* FOXP3* in thymus of C57BL/6 and BALB/c mice inoculated with 10.000 LD_50_ of YFV17D. RQ = relative quantification representative of geometric mean values of 2^−ΔΔCt^. RQ values expressed as fold change relative to control group values (baseline = 1). Statistical analysis was performed using the nonparametric test Mann-Whitney two-tailed. Values close to the level of significance (*P* < 0.05) are marked with *∗*.

Interestingly, we observed an increased expression* FOXP3* in liver of YFV17D-treated C57BL/6 mice compared to thymus and spleen (*P* < 0.05) (Figure 5(c)). No differences in* FOXP3* transcript levels among tissues were observed in BALB/c mice (Figure 5(d)). Increased transcript levels of* H2-Q10* (Q10) were observed in the liver of YFV17D-treated C57BL/6 mice compared to thymus and spleen (*P* < 0.05) (Supplemental data III). Noteworthy,* H2-Q10* is a murine MHC Ib gene encoding the Q10 molecule, expressed mainly in the liver and related to the regulation of immune responses [73, 74]. Finally, compared to BALB/c mice, the C57BL/6 thymus showed significant higher transcript levels for* H2-T23* (Qa-1/HLA-E) in all harvested days a.i. (*P* < 0.05) and* FOXP3* at Day 5 a.i. (Figures 6(a) and 6(b)). No differences were found for the other genes between both strains.

We speculate that the increased expression of* H2-T23 (Qa-1/HLA-E)*,* H2-Q10 (Q10),* and* FOXP3* in YFV17D-immunized C57BL/6 mice may contribute to the establishment of a regulatory network that protects the YFV17D-infected cells from immune system, since both molecules are clearly involved in the modulation of the immune system and the generation of regulatory T cells, thus, reflecting in an inability of viral load control and in a greater susceptibility of this strain to YFV17D infection. In BALB/c mice, on the other hand, the observation of liver and spleen hypertrophy, more pronounced signs of infection, which may be indicative of more robust antiviral immune response, better control of viral load, together with lower transcription of immunomodulatory genes in thymus and peripheral tissues in YFV17D-immunized mice compared to C57BL/6, may contribute to a better resistance to YFV17D infection.

Among the several factors involved in the onset of autoimmunity, not only viral infections but also live virus attenuated vaccines are potentially relevant to development of autoimmune disorders [42–44]. The thymus is the primary organ involved in induction and maintenance of self-tolerance in T lymphocytes, particularly due to Aire activity and generation of natural regulatory T cells [2, 66]. Moreover, the expression of immunomodulatory molecules, such as those from MHC Ib, is essential to ensure maintenance of self-tolerance in potentially autoreactive T lymphocyte clones in peripheral tissues [11, 16, 35, 38]. Alterations in thymic function and in MHC Ib expression could change the regulation of immune responses permitting the onset of autoimmune disorders.

Besides the fact that the YFV17D vaccine is the only preventive measure against the hemorrhagic fever caused by YFV, studies involving the biological effects after immunization with YFV17D are relevant since several evidences show the emergence of autoimmune disorders in patients after YFV17D immunization [45, 46]. Additionally, the contraindication of YFV17D vaccine in individuals with thymic diseases [29], immunosuppressed, or with rheumatic diseases, such as lupus erythematosus, rheumatoid arthritis, and polymyalgia rheumatica [75], leads to speculation about the influence of YFV17D immunization in thymus, particularly in the immunoregulatory mechanisms of tolerance.

## 4. Conclusions

The present study showed that YFV17D immunization influences the transcriptional profiles of immunomodulatory genes in thymus by promoting the induction of MHC Ib genes:* H2-Q7* (Qa-2/HLA-G) and* H2-T23* (Qa-1/HLA-E), and the repression of* AIRE* and* FOXP3,* indicating a possible influence on thymic function. Also, we highlighted the thymus as a target organ for YFV17D virus and as a main site of expression of MHC Ib during YFV17D infection. Similarly, we demonstrated the relevance of the genetic background of C57BL/6 and BALB/c mice strains in tolerance mechanisms during YFV17D infection, suggesting that increased expression of* H2-T23* (Qa-1/HLA-E) and* FOXP3* transcripts in thymus and liver of C57BL/6 mice, together with a defective control of viral load, might be indicative of a higher susceptibility of this strain to YFV17D infection. The present work may contribute to further studies involving the thymus and its immunomodulatory molecules in the maintenance of central and peripheral tolerance during antiviral immune responses.

## Supplementary Material

The morphological analysis of internal organs in YFV17D - immunized animals revealed liver and spleen hypertrophy at Day 7 a.i in BALB/c mice compared to C57BL/6 and compared to control groups. (Supplemental data I). The gene expression analysis of H2-Q10(Qa10) in thymus of C57BL/6 and BALB/c mice inoculated with 10.000 LD_50_ of YFV17D or PBS showed no differences in H2-Q10(Qa10) transcription compared to the control group. (Supplemental data II). Increased transcript levels of H2-Q10(Q10) were observed in the liver of YFV17D-treated C57BL/6 mice compared to thymus and spleen (P<0.05). (Supplemental data III).
