# Supplementary material for: The Attenuated Live Yellow Fever Virus 17D Infects the Thymus and Induces Thymic Transcriptional Modifications of Immunomodulatory Genes in C57BL/6 and BALB/C Mice
Source: Autoimmune Dis. 2015 Sep 17;2015:503087. doi: 10.1155/2015/503087 (PMC4589579; doi:10.1155/2015/503087)
Supplement: Supplementary file 1 — The morphological analysis of internal organs in YFV17D - immunized animals revealed liver and spleen hypertrophy at Day 7 a.i in BALB/c mice compared to C57BL/6 and compared to control groups. (Supplemental data I). The gene expression analysis of H2-Q10(Qa10) in thymus of C57BL/6 and BALB/c mice inoculated with 10.000 LD50 of YFV17D or PBS showed no differences in H2-Q10(Qa10) transcription compared to the control group. (Supplemental data II). Increased transcript levels of H2-Q10(Q10) were observed in the liver of YFV17D-treated C57BL/6 mice compared to thymus and spleen (P<0.05). (Supplemental data III). [file 503087.f1.pdf]

# Supplemental Data

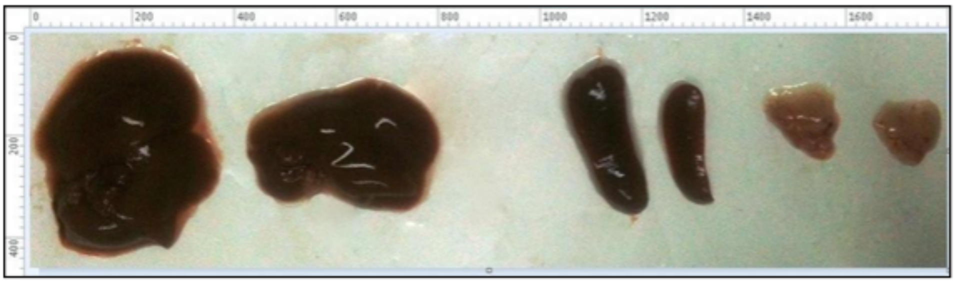

**I**: **Visual inspection of Liver, Spleen and Thymus of BALB/c (Left) and C57BL/6 (right) 7 days after inoculation of 10,000 $LD_{50}$ of yellow fever virus (YFV17D). Representative of at least 5 independent samples.**

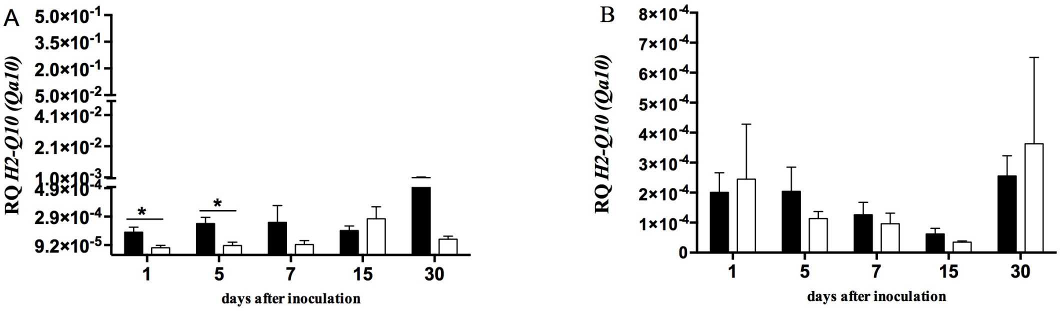


**II - Gene expression of *H2-Q10*(Qa10) in thymus of (A) C57BL/6 and (B) BALB/c mice inoculated with 10.000 $LD_{50}$ of YFV17D or PBS.** RQ = Relative Quantification representative of geometric mean values of $2^{-\Delta\Delta Ct}$. Tissue samples were obtained in triplicate from different animals for each treatment. Each experiment was independently performed at least three times. Statistical analysis was performed using the non-parametric test Mann-Whitney two-tailed. Values close to the level of significance (*P<0.05*) are marked with (*).

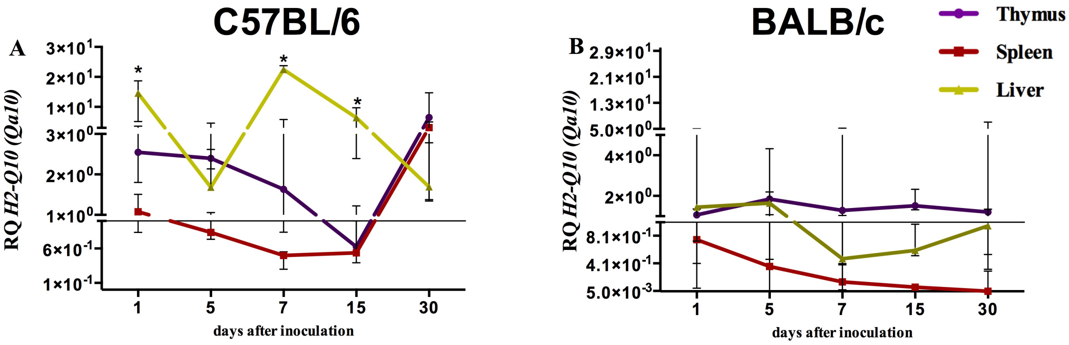

**III - Transcriptional profiles of *H2-Q10*Qa-10) in harvested tissues of (A) C56BL/6 and (B) BALB/c mice inoculated with 10.000 $LD_{50}$ of YFV17D.** RQ = Relative Quantification representative of geometric mean values of $2^{-\Delta\Delta Ct}$. RQ values expressed as fold change relative to control group values (baseline = 1). Statistical analysis was performed using the non-parametric test Kruskal-Wallis Dunn's multiple comparison. Values close to the level of significance ($P<0.05$) are marked with (*).
